# The Prevalence of Autism in the Criminal Justice System: A Systematic Review

**DOI:** 10.1192/bjo.2022.179

**Published:** 2022-06-20

**Authors:** Verity Chester, Karen Bunning, Samuel Tromans, Regi Alexander, Peter Langdon

**Affiliations:** 1 University of East Anglia, Norwich, United Kingdom; 2 Hertfordshire Partnership University NHS Foundation Trust, Norwich, United Kingdom; 3 University of Leicester, Leicester, United Kingdom; 4 Leicestershire Partnership NHS Trust, Leicester, United Kingdom; 5 University of Hertfordshire, Hatfield, United Kingdom; 6 University of Warwickshire, Coventry, United Kingdom

## Abstract

**Aims:**

Autism prevalence is currently estimated to be approximately 1%. Ascertaining autism prevalence within the Criminal Justice System (CJS) has implications for understanding clinical and forensic need, alongside facilitating autism-specific CJS responses. This review aims to systematically identify and synthesise studies that investigate autism prevalence within CJS cohorts, and CJS involvement in autistic cohorts.

**Methods:**

A systematic review of published studies that investigated autism prevalence within the CJS. A systematic search of major online databases was conducted in November 2021, including the ancestry method/expert consultation. Studies were qualitatively analysed, with reporting quality appraised.

**Results:**

The search yielded 6491 articles. Following duplicate removal, 2942 articles remained for screening, of which 2857 did not meet inclusion criteria. Therefore, full texts of 85 articles were accessed, and 34 qualified for inclusion.

Prevalence rates of autism in the CJS were examined in 19 studies, 12 focused on forensic settings (e.g. secure psychiatric services/prisons/court), with 7 focused on forensic psychiatric assessment referrals. Prevalence rates of autistic people within the CJS reported by the included studies varied from 1–60%. This variation appeared related to factors such as the characteristics of the forensic setting/cohort, the method of autism screening/diagnosis), and whether participants had co-occurring intellectual disabilities.

Prevalence rates of CJS involvement in autistic populations were examined in 15 studies, with reported rates varying by 3–48%, with variation appearing related to a lack of cohesion in the definition of CJS involvement, with focus on variables including self-reported offending behaviour, police contact, or criminal convictions. These studies reported rates of offending by autistic people at a rate equivalent to, or lower than the general population/comparison sample.

**Conclusion:**

Studies examining prevalence of CJS involvement among autistic people indicate a rate of offending at a lower, or equivalent level to the general population or comparison samples. However, studies examining prevalence of autistic people within CJS settings suggest they are over-represented. Possible explanations fall within three categories:

- pre-sentencing CJS factors - e.g. autistic people being more likely to be caught for their criminal behaviour, to confess during police interviews, to enter a guilty plea, or to have difficulty advocating for their rights in court
- autistic offender factors – whether autistic people who do engage in criminal behaviour, engage in behaviour of a higher severity, possibly reflecting high rates of comorbid mental disorder
- post-sentencing CJS factors – whether autistic people who offend are sentenced more harshly, or the possibility that a lack of autism sensitive forensic rehabilitative programmes and risk assessments may contribute to longer stays within forensic settings.

